# Supplementary material for: City-wide built environment SARS-CoV-2 detection for COVID-19 surveillance
Source: PLOS Glob Public Health. 2025 Sep 8;5(9):e0004306. doi: 10.1371/journal.pgph.0004306 (PMC12416635; doi:10.1371/journal.pgph.0004306)
Supplement: S1 Text — S1 Table A. Descriptive statistics by neighborhood. S2 Table B. Correlation coefficients between geometric means of viral copies and hospital COVID-19 admitted patient census or regional wastewater signal, by setting. S1 Table C. Performance estimates of random intercepts linear models for weekly COVID-19-linked hospital census, evaluated by 5-fold cross-validation repeated 10 times, with predictive performance expressed in terms of root mean squared error (RMSE) and R2. S1 Table D. Coefficients, standard errors, and other information pertaining to finalized mixed effects linear models for weekly hospital census with each or both surface-detection variables as predictors (models which were evaluated by cross-validation in Supplemental Table 3). S1 Table E. Spearman and Pearson correlation tests from surface detection, waste-water surveillance and hospital census variables. S1 Fig A. Neighborhoods evaluated in this study. S1 Fig B. Geometric mean of viral copies over time by population. S1 Fig C. Neighborhood-level aggregated SARS-CoV-2 positivity and viral copies over time. (PDF) [file pgph.0004306.s001.pdf]

S1. SUPPLEMENTARY MATERIALS

SUPPLEMENTAL TABLES

S1. Table A. Descriptive statistics by neighborhood. 'Viral copies' refers to the weekly log_{10}-transformed geometric mean number of viral copies plus one copy.

Area	Swabs (n)	Positivity (95% CI)	Viral Copies (95% CI)	Start Date	End Date	Sites (N)
Neighborhood-1	418	46% (41-51)	2.82 (2.49-3.19)	2022-11-03	2023-03-30	3
Neighborhood-2	100	20% (13-29)	1.43 (1.23-1.66)	2022-11-09	2023-03-30	1
Neighborhood-3	336	50% (45-55)	4.72 (3.84-5.8)	2022-11-09	2023-03-30	3
Neighborhood-4	348	54% (48-59)	4.96 (4.11-5.99)	2022-11-11	2023-03-30	3
Neighborhood-5	424	41% (36-46)	2.88 (2.49-3.33)	2022-10-25	2023-03-30	3
Neighborhood-6	237	45% (39-51)	2.97 (2.47-3.58)	2022-11-09	2023-03-30	3

S1. Table B. Correlation coefficients between geometric means of viral copies and hospital COVID-19 admitted patient census or regional wastewater signal, by setting. Viral copies refers to the weekly $\log_{10}$-transformed geometric mean number of viral copies plus one copy.

Variables	Spearman r (p-value)	Pearson r (p-value)
Swab viral copies ~ hospital census	0.643 (1.7×10^{-3})	0.728 (1.9×10^{-4})
Swab viral copies ~ regional waste-water	-0.004 (0.99)	0.074 (0.8)

S1. Table C. Performance estimates of random intercepts linear models for weekly COVID-19-linked hospital census, evaluated by 5-fold cross-validation repeated 10 times, with predictive performance expressed in terms of root mean squared error (RMSE) and R^2. Viral copies represent the weekly $\log_{10}$-transformed geometric mean number of viral copies plus one copy.

**Fixed Effect Specification**	**RMSE (patients)**	**R^2**
Positivity	13.73 ± 0.25	0.133 ± 0.011
Viral copies	12.57 ± 0.26	0.417 ± 0.017
Positivity + Viral copies	12.41 ± 0.27	0.403 ± 0.021

S1. Table D. Coefficients, standard errors, and other information pertaining to finalized mixed effects linear models for weekly hospital census with each or both surface-detection variables as predictors (models which were evaluated by cross-validation in Supplemental Table 3). All models included the same number of observations (N=77 weekly observations) from 5 groups (ie. a random intercept for each hospital). Uncertainty intervals (equal-tailed) and p-values (two-tailed) computed using a Wald t-distribution approximation. Uncertainty intervals for random effect variances computed using a Wald z-distribution approximation. Weekly viral copies values were computed as the log_{10}-transformed geometric mean of the number of viral copies plus one copy (to avoid undefined values for negative tests).

Model Predictors	Fixed or Random Effect	Parameter	Coef	SE	95% CI	t (df)	p
Viral copies	Fixed	Intercept	13.2	6.72	-0.2, 26.59	1.96 (73)	0.053
	Fixed	Viral copies	4.12	2.03	0.09, 8.16	2.04 (73)	0.045
	Random	SD (Intercept: site)	13.45	4.85	6.64, 27.26	-	-
	Random	SD (Residual)	5.95	0.50	5.05, 7.01	-	-
Positivity	Fixed	Intercept	19.93	8.68	2.63, 37.22	2.30 (73)	0.025
	Fixed	Positivity	-0.87	5.95	-12.73,10.99	-0.15 (73)	0.88
	Random	SD (Intercept: site)	14.76	5.29	7.31, 29.82	-	-
	Random	SD (Residual)	6.09	0.51	5.16, 7.17	-	-
Positivity + viral copies	Fixed	Intercept	21.27	8.1	5.13, 37.42	2.63 (72)	0.011
	Fixed	Viral copies	6.82	2.52	1.8, 11.84	2.71	0.008
	Fixed	Positivity	-12.73	7.23	-27.14, 1.68	-1.76	0.082
	Random	SD (Intercept: site)	13.4	4.83	6.61, 27.15	-	-
	Random	SD (Residual)	5.86	0.5	4.97, 6.92	-	-

S1. Table E. Spearman and Pearson correlation tests from surface detection, waste-water surveillance and hospital census variables. Correlations between environmental detection variables with lagging outcomes were also tested where noted (e.g.: *Admits n weeks later*). Subgroups of environmental detection measures are indicated where values were taken from hospitals, schools, libraries, or schools and libraries, as opposed to from all sites. Variable names have been abbreviated as follows: “Census” represent the total number of COVID-19-related hospital census across the five hospitals monitored by surface sampling; “Copies” represents the weekly log_{10}-transformed geometric mean number of viral copies plus one copy; “WW” represents the weekly mean value of SARS-CoV-2 N1 and N2 targets, relative to PPMoV.

Environmental detection	Outcome	Spearman		Pearson	
		**r**	**p-value**	**r**	**p-value**
Copies: Hospital	Census	0.76	9.5e-05	0.84	3.9e-06
Copies: Hospital	Census 1 week later	0.65	0.0025	0.59	0.0073
Copies: Hospital	Census 2 weeks later	0.56	0.016	0.53	0.022
Copies: Library	Census	-0.14	0.57	-0.3	0.21
Copies: Library	Census 1 week later	-0.16	0.51	-0.28	0.25
Copies: Library	Census 2 weeks later	-0.18	0.47	-0.27	0.29
Copies: School	Census	-0.58	0.0092	-0.37	0.12
Copies: School	Census 1 week later	-0.66	0.003	-0.45	0.061
Copies: School	Census 2 weeks later	-0.77	0.00028	-0.61	0.0097
Copies: All sites	Census	0.64	0.0017	0.73	0.00019
Copies: All sites	Census 1 week later	0.49	0.03	0.55	0.012
Copies: All sites	Census 2 weeks later	0.34	0.16	0.31	0.19
Copies: Schools & Libraries	Census	-0.41	0.065	-0.44	0.046
Copies: Schools & Libraries	Census 1 week later	-0.49	0.028	-0.48	0.033
Copies: Schools & Libraries	Census 2 weeks later	-0.58	0.0091	-0.53	0.019
Positivity: Hospital	Census	0.54	0.013	0.59	0.0066
Positivity: Hospital	Census 1 week later	0.4	0.087	0.47	0.044
Positivity: Hospital	Census 2 weeks later	0.31	0.21	0.35	0.16
Positivity: Library	Census	-0.25	0.3	-0.4	0.084
Positivity: Library	Census 1 week later	-0.27	0.27	-0.3	0.21
Positivity: Library	Census 2 weeks later	-0.37	0.13	-0.4	0.099
Positivity: School	Census	-0.53	0.019	-0.52	0.022
Positivity: School	Census 1 week later	-0.66	0.0028	-0.63	0.0047
Positivity: School	Census 2 weeks later	-0.76	0.00035	-0.74	0.00076
Positivity: All sites	Census	0.13	0.59	0.021	0.93
Positivity: All sites	Census 1 week later	-0.16	0.5	-0.21	0.38
Positivity: All sites	Census 2 weeks later	-0.43	0.067	-0.46	0.045
Positivity: Schools & Libraries	Census	-0.47	0.03	-0.56	0.0079
Positivity: Schools & Libraries	Census 1 week later	-0.55	0.012	-0.6	0.0051
Positivity: Schools & Libraries	Census 2 weeks later	-0.63	0.0041	-0.64	0.003
WW	Census	-0.15	0.51	-0.075	0.73
WW	Census 1 week later	-0.089	0.69	0.041	0.86
WW	Census 2 weeks later	-0.067	0.77	0.086	0.71
WW 1 week earlier	Census 2 weeks later	-0.071	0.77	0.0075	0.97
WW 1 week later	Census	-0.22	0.33	-0.2	0.36
WW 1 week later	Census 1 week later	-0.23	0.29	-0.13	0.56
WW 1 week later	Census 2 weeks later	-0.21	0.37	-0.012	0.96
Copies: Hospital	WW	-0.11	0.65	-0.077	0.75
Copies: Hospital	WW 1 week ago	-0.021	0.93	0.016	0.95
Copies: Hospital	WW 1 week later	-0.051	0.84	-0.16	0.51
Copies: Library	WW	0.2	0.4	0.079	0.74

Environmental detection	Outcome	Spearman		Pearson	
		r	p-value	r	p-value
Copies: Library	WW 1 week ago	0.5	0.026	0.38	0.097
Copies: Library	WW 1 week later	-0.065	0.79	-0.2	0.41
Copies: School	WW	0.3	0.21	0.21	0.38
Copies: School	WW 1 week ago	0.35	0.14	0.28	0.25
Copies: School	WW 1 week later	0.19	0.45	-0.037	0.88
Copies: All sites	WW	-0.0039	0.99	0.074	0.75
Copies: All sites	WW 1 week ago	0.29	0.21	0.22	0.34
Copies: All sites	WW 1 week later	-0.18	0.45	-0.14	0.56
Copies: Schools & Libraries	WW	0.3	0.18	0.14	0.54
Copies: Schools & Libraries	WW 1 week ago	0.45	0.038	0.32	0.16
Copies: Schools & Libraries	WW 1 week later	0.14	0.56	-0.08	0.74
Positivity: Hospital	WW	-0.26	0.26	-0.15	0.53
Positivity: Hospital	WW 1 week ago	-0.12	0.61	-0.088	0.71
Positivity: Hospital	WW 1 week later	-0.21	0.39	-0.13	0.61
Positivity: Library	WW	0.25	0.29	0.17	0.47
Positivity: Library	WW 1 week ago	0.59	0.0057	0.49	0.027
Positivity: Library	WW 1 week later	-0.021	0.93	-0.12	0.61
Positivity: School	WW	0.26	0.29	0.18	0.47
Positivity: School	WW 1 week ago	0.37	0.12	0.24	0.32
Positivity: School	WW 1 week later	0.14	0.59	-0.067	0.79
Positivity: All sites	WW	0.15	0.51	0.088	0.71
Positivity: All sites	WW 1 week ago	0.41	0.066	0.32	0.16
Positivity: All sites	WW 1 week later	0.11	0.64	-0.096	0.69
Positivity: Schools & Libraries	WW	0.26	0.26	0.15	0.51
Positivity: Schools & Libraries	WW 1 week ago	0.45	0.04	0.32	0.15
Positivity: Schools & Libraries	WW 1 week later	0.11	0.64	-0.056	0.81

SUPPLEMENTAL FIGURES

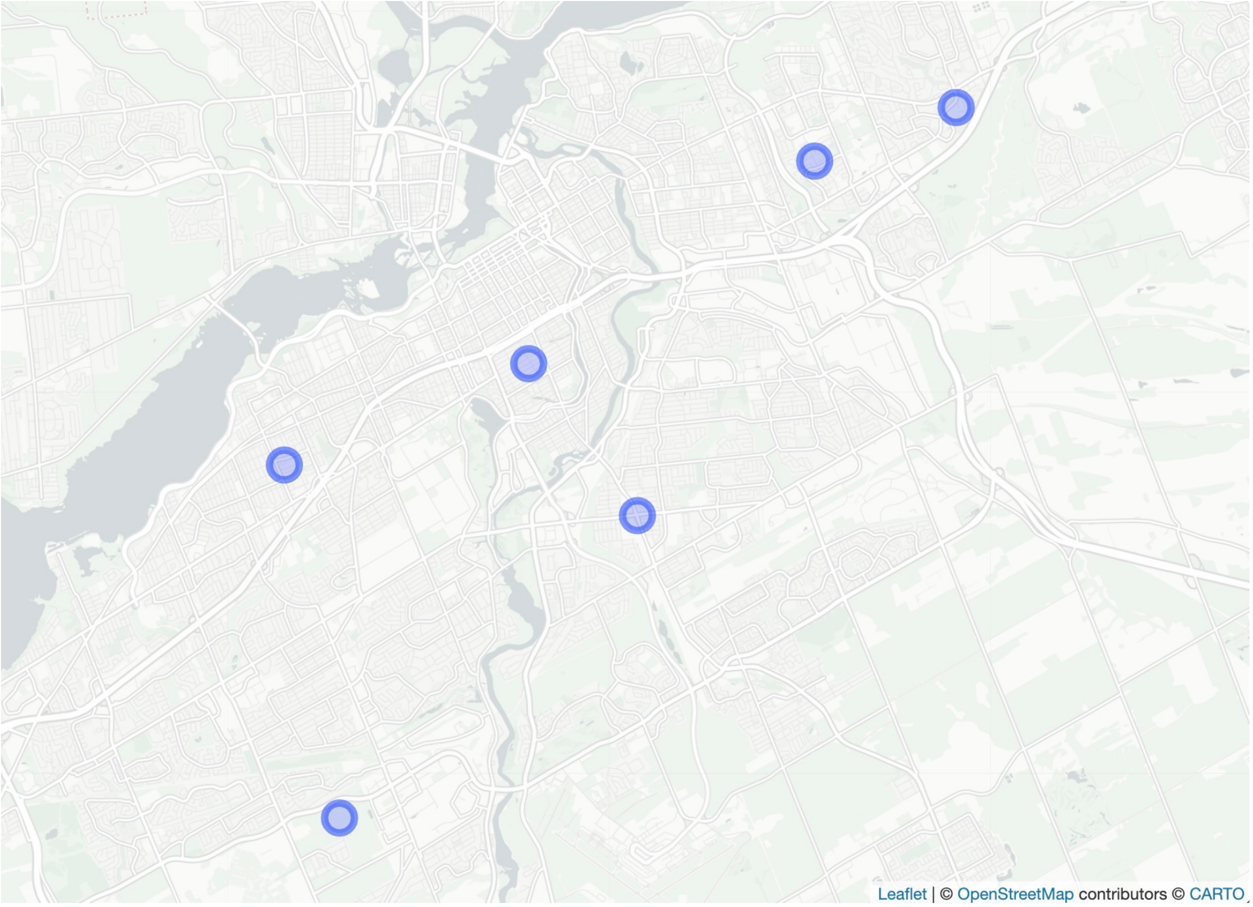

S1. Fig A. Neighborhoods evaluated in this study.

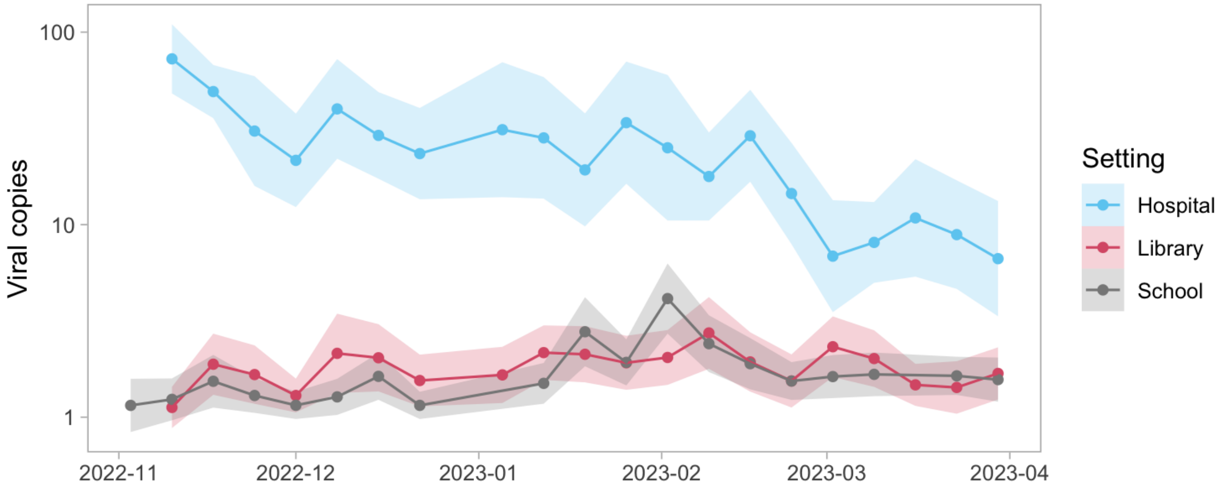

S1. Fig B. Geometric mean of viral copies over time by population.

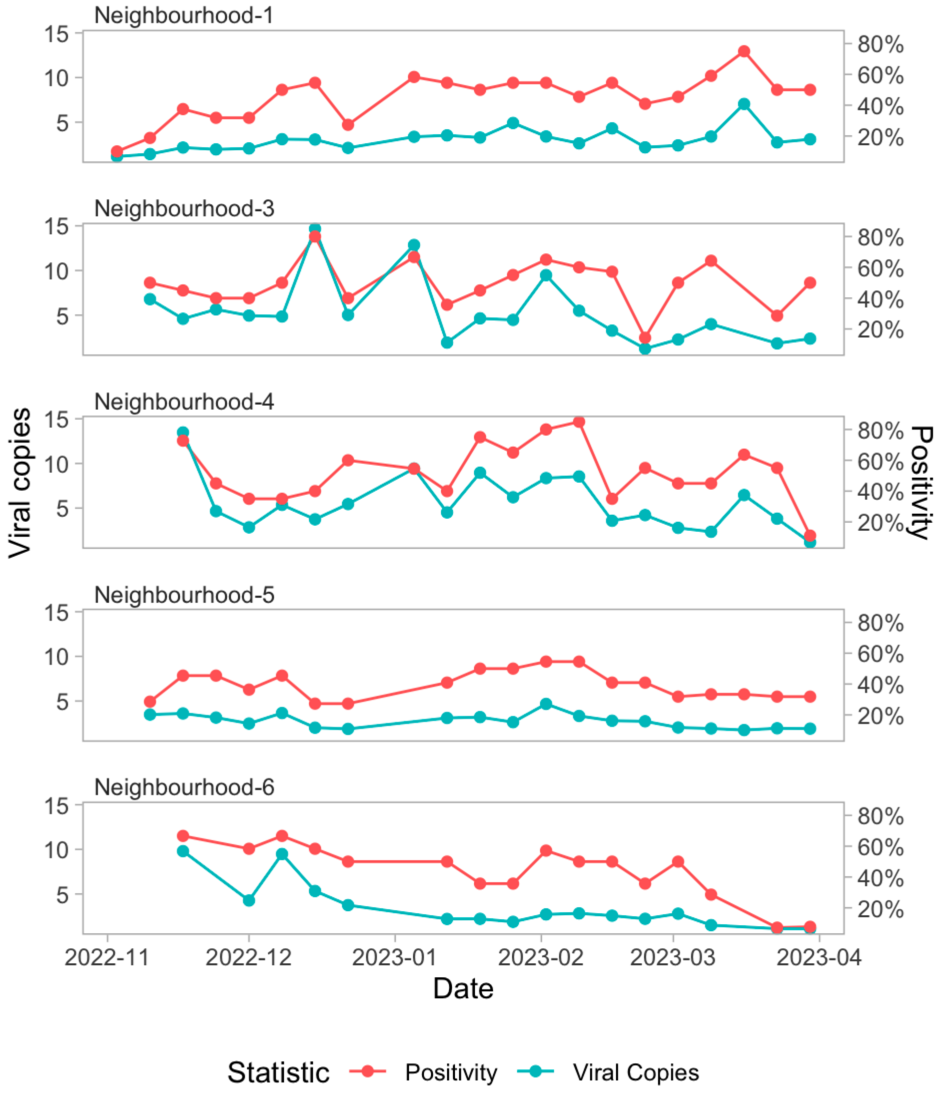

S1. Fig C. Neighborhood-level aggregated SARS-CoV-2 positivity and viral copies over time.
